# Application of machine learning to pretherapeutically estimate dosimetry in men with advanced prostate cancer treated with ^177^Lu-PSMA I&T therapy

**DOI:** 10.1007/s00259-022-05883-w

**Published:** 2022-06-30

**Authors:** Song Xue, Andrei Gafita, Chao Dong, Yu Zhao, Giles Tetteh, Bjoern H. Menze, Sibylle Ziegler, Wolfgang Weber, Ali Afshar-Oromieh, Axel Rominger, Matthias Eiber, Kuangyu Shi

**Affiliations:** 1 Dept. Nuclear Medicine, Inselspital, Bern University Hospital, University of Bern, Bern, Switzerland; 2 Dept. Nuclear Medicine, Technical University of Munich, Munich, Germany; 3 Dept. Molecular & Medical Pharmacology, University of California, Los Angeles, CA USA; 4 Dept. Electrical Engineering, Technical University of Munich, Munich, Germany; 5 Dept. Informatics, Technical University of Munich, Munich, Germany; 6 Dept. Nuclear Medicine, University Hospital, LMU Munich, Munich, Germany

**Keywords:** Radioligand therapy, ^177^Lu-PSMA I&T, Dosimetry, Treatment planning, Machine learning

## Abstract

**Purpose:**

Although treatment planning and individualized dose application for emerging prostate-specific membrane antigen (PSMA)-targeted radioligand therapy (RLT) are generally recommended, it is still difficult to implement in practice at the moment. In this study, we aimed to prove the concept of pretherapeutic prediction of dosimetry based on imaging and laboratory measurements before the RLT treatment.

**Methods:**

Twenty-three patients with metastatic castration-resistant prostate cancer (mCRPC) treated with ^177^Lu-PSMA I&T RLT were included retrospectively. They had available pre-therapy ^68^ Ga-PSMA-HEBD-CC PET/CT and at least 3 planar and 1 SPECT/CT imaging for dosimetry. Overall, 43 cycles of ^177^Lu-PSMA I&T RLT were applied. Organ-based standard uptake values (SUVs) were obtained from pre-therapy PET/CT scans. Patient dosimetry was calculated for the kidney, liver, spleen, and salivary glands using Hermes Hybrid Dosimetry 4.0 from the planar and SPECT/CT images. Machine learning methods were explored for dose prediction from organ SUVs and laboratory measurements. The uncertainty of these dose predictions was compared with the population-based dosimetry estimates. Mean absolute percentage error (MAPE) was used to assess the prediction uncertainty of estimated dosimetry.

**Results:**

An optimal machine learning method achieved a dosimetry prediction MAPE of 15.8 ± 13.2% for the kidney, 29.6% ± 13.7% for the liver, 23.8% ± 13.1% for the salivary glands, and 32.1 ± 31.4% for the spleen. In contrast, the prediction based on literature population mean has significantly larger MAPE (*p* < 0.01), 25.5 ± 17.3% for the kidney, 139.1% ± 111.5% for the liver, 67.0 ± 58.3% for the salivary glands, and 54.1 ± 215.3% for the spleen.

**Conclusion:**

The preliminary results confirmed the feasibility of pretherapeutic estimation of treatment dosimetry and its added value to empirical population-based estimation. The exploration of dose prediction may support the implementation of treatment planning for RLT.

**Supplementary Information:**

The online version contains supplementary material available at 10.1007/s00259-022-05883-w.

## Introduction

Radioligand therapy (RLT) is a contemporary approach to radiation oncology, aiming to deliver the maximal destructive radiation dose via cancer-targeting radiopharmaceutical. Radioactive ligands for the prostate-specific membrane antigen (PSMA) have emerged for the treatment of metastatic castration-resistant prostate cancer (mCRPC) [1–3]. Notably, ^177^Lu-PSMA-617 was validated recently in a phase III randomized clinical trial [4] which led to the U.S. Food and Drug Administration (FDA) approval [5].

Despite the early success of RLT, concerns have been raised about the risks of inadequate trade-off between therapeutic dose and side effects. Currently, the protocols for administering the radiopharmaceuticals are assessed on a population basis, and the activity to administer was determined for a specific patient group based on preceding studies [6]. However, the European Council Directive (2013/59 Euratom) mandates that RLT treatments should be planned according to the optimal radiation dose tailored for individual patients, as has long been the case for external beam radiotherapy (EBRT) or brachytherapy [7, 8]. An essential requirement of RLT treatment planning is to estimate the absorbed dose in advance of therapy [9, 10].

Prior knowledge of the biodistribution of the therapeutic agent via the pre-therapy imaging assists to optimize the trade-off between tumor destruction and irradiation of healthy tissues [11–15]. Concepts, such as physiologically based pharmacokinetic (PBPK) modeling, have been proposed to estimate the spatiotemporal pharmacokinetics of imaging agents and then extrapolate to the treatment agents [7, 10, 16, 17]. Normal organ and tumor pharmacokinetics can be assessed by a series of cross-sectional whole-body SPECT scans [18]. However, these require a large amount imaging time and are often not feasible in routine clinical practice. An alternative is pharmacokinetic modeling based on the activity concentration in the blood and a computational model which describes the binding of the ligand to its target as well as its metabolism and excretion. However, these models can be numerically unstable [18], thus presenting a dilemma in making a trade-off between numerical stability and physiological fidelity [19]. Due to these technical limitations, the pre-therapy imaging is usually only used in current practice to qualitatively select candidates for RLT and to rule out obvious risks. The patients are still treated with a fixed radiopharmaceutical activity and fraction interval protocol [20, 21].

**Fig. 1:**
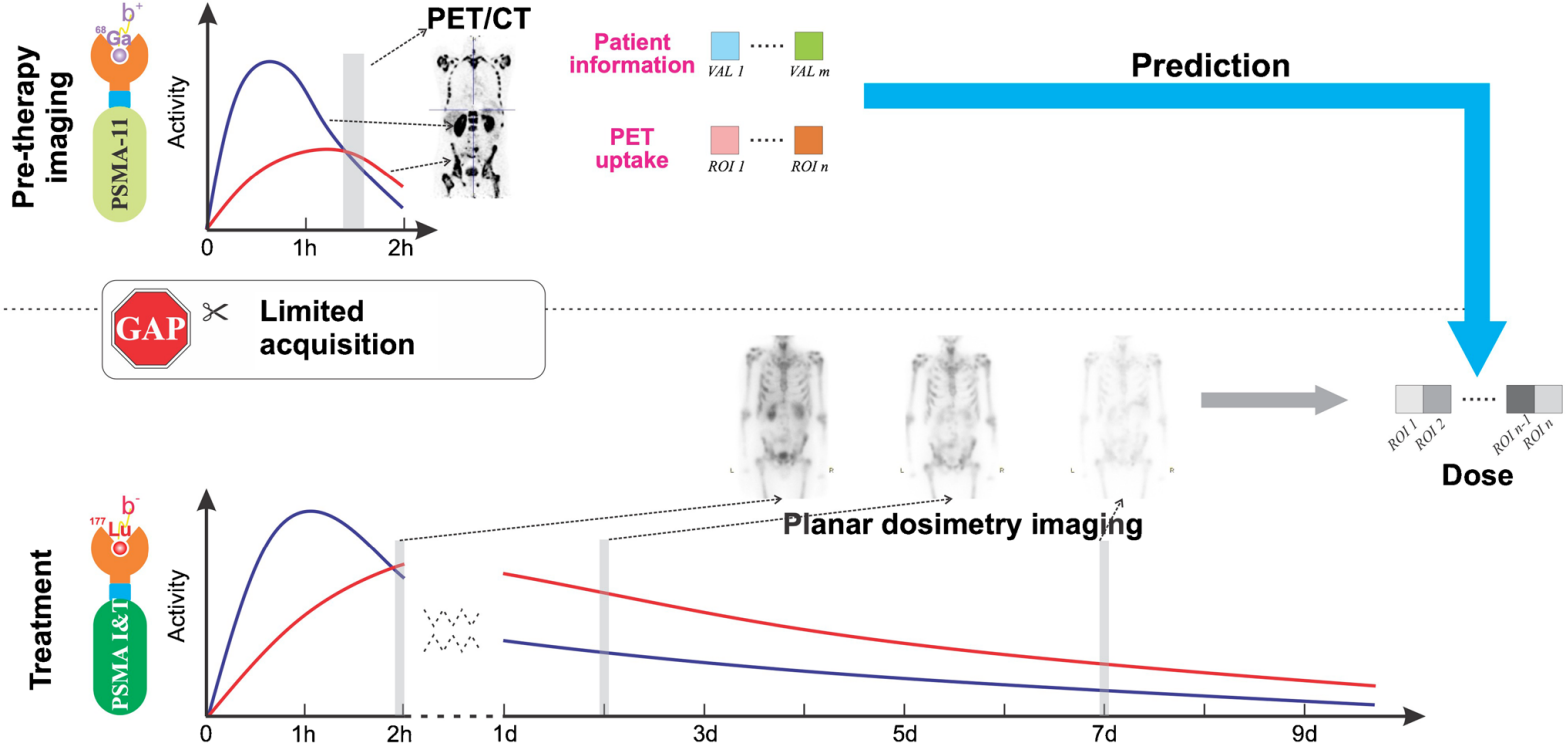
Illustration of our proposed method; our study aims to prove the concept of individual dosimetry prediction based on pre-therapy imaging and laboratory measurements, by providing an alternative solution with machine learning (ML) technique

As illustrated in Fig. 1, our study aimed to prove the concept of dosimetry prediction based on pre-therapy PET imaging and blood test results by providing an alternative solution with machine learning (ML) technique. We aimed to evaluate the feasibility to obtain estimation of the pharmacokinetics of PSMA ligands over several days from a single time point PET scan acquired after 1 h. Such concept would help to avoid a series of whole-body imaging with long procedure scans. Furthermore, it could be directly implemented in the current routine clinical protocol, which may provide a practical solution for dosimetry-based treatment planning for RLT.

## Materials and methods

### Patient cohorts

Patients with metastatic castration-resistant prostate cancer (mCRPC) who (i) were treated with ^177^Lu-PSMA I&T RLT at Klinikum rechts der Isar between December 2014 and August 2017, (ii) received pretherapeutic ^68^ Ga-PSMA-HEBD-CC (PSMA-11) PET/CT within 2 months of treatment initiation, and (iii) received at least 3 post-therapeutic planar imaging in conjunction with 1 SPECT/CT were screened retrospectively for inclusion. Twenty-three patients met the eligibility criteria and were included. After image review, 1 patient with invisible liver and spleen on planar PET scan was excluded. Overall, 43 cycles of ^177^Lu-PSMA I&T RLT were applied (21 first, 11 s, 5 third, and 6 fourth or further cycles). For each cycle, the patients referred to ^68^ Ga-PMSA-HEBD-CC PET/CT for pre-therapy imaging, and less than 2 months later underwent 3–5 planar whole-body scans and SPECT/CT after injecting approximately 7.4 GBq (7.3 ± 0.3 GBq) ^177^Lu-PSMA I&T, for the purpose of dosimetry investigation. For all subjects, anterior and posterior whole-body scintigraphy was performed at least at three time points, which are 30–150 min, 24 h, and 6–8 days after injection. The retrospective analysis was conducted in accordance with the requirements of the respective local ethics committees in Germany, the institutional review board (IRB) of the Technische Universität München approved this study (IRB reference no: 115/18). All patients signed written informed consent prior to the use of Lu-PSMA as part of a compassionate use application. ^177^Lu-PSMA I&T was administered in compliance with The German Medicinal Products Act, AMG §13 2b, and in accordance with the responsible regulatory body (Government of Oberbayern).

### Image analysis and dosimetry estimation

Our proposed ML-based dose estimation method was designed as a supervised learning approach, which requires labeled training datasets. As shown in Fig. 1, both clinical information of patients and PET imaging data are the inputs to the model, and the corresponding organ-level dosimetry is used as the ground truth for training.

As shown in Table 1, both quantities derived from pre-therapy imaging and blood test results were collected as input for the development of the prediction model. Among them, 11 blood tests results were included such as creatinine, albumin, and lactate dehydrogenase (LDH). As for the features from PET imaging, targeted organs (whole body, kidneys, spleen, liver, parotid glands, and submandibular glands) were delineated manually and reviewed by board-certified nuclear medicine physicians. Imaging parameters including volume-related features and voxel intensity-related features, as well as standard uptake value (SUV)-related features, were calculated from the targeted organs.

**Table 1:** Recruited clinical features (blood test) and PET features (volume, voxel intensity, and SUV) for the development of our proposed machine learning algorithm

Type of feature	Name of feature	Description
Volume**-**related features	Vol	Volume of targeted organ
	V40	Percentage volume with at least 40% intensity
	V70	Percentage volume with at least 70% intensity
	V90	Percentage volume with at least 90% intensity
Voxel intensity**-**related features	Total Voxel	Total amount of voxel
	Mean	Mean intensity value
	Min	Minimum intensity value
	Max	Maximum intensity value
	Sum	Summation of intensity value
	Std. Dev	Standard deviation of intensity value
	Skewness	Measure of the symmetry of the intensity distribution
	Kurtosis	Measure of the shape of the peak of the intensity distribution
	Median	Median intensity value
SUV**-**related features	SUV peak	Average activity concentration within a 1 cm^3^ spherical VOI centered on the “hottest focus” within the tumor image multiplied by the ratio of lean body mass (LBM) to injected activity decayed to time of scan
	SUV mean	Mean SUV value
	SUV min	Minimum SUV value
	SUV max	Maximum SUV value
	SUV TLG	The product of SUV mean and metabolic tumor volume (MTV)
	SUV std. Dev	Standard deviation of SUV value
	SUV median	Median SUV value
Blood tests	Interval	
	Creatinine clearance (ml/min)	
	Alkaline phosphate (ALP) (U/L)	
	Total bilirubin (mg/dL)	
	Lactate dehydrogenase (LDH) (U/L)	
	Albumin (g/L)	
	Prothrombin time (min)	
	Leukocyte count (/L)	
	Hemoglobin (g/L)	
	Thrombocyte count (/L)	
	PSA (μg/L)	

**Fig. 2:**
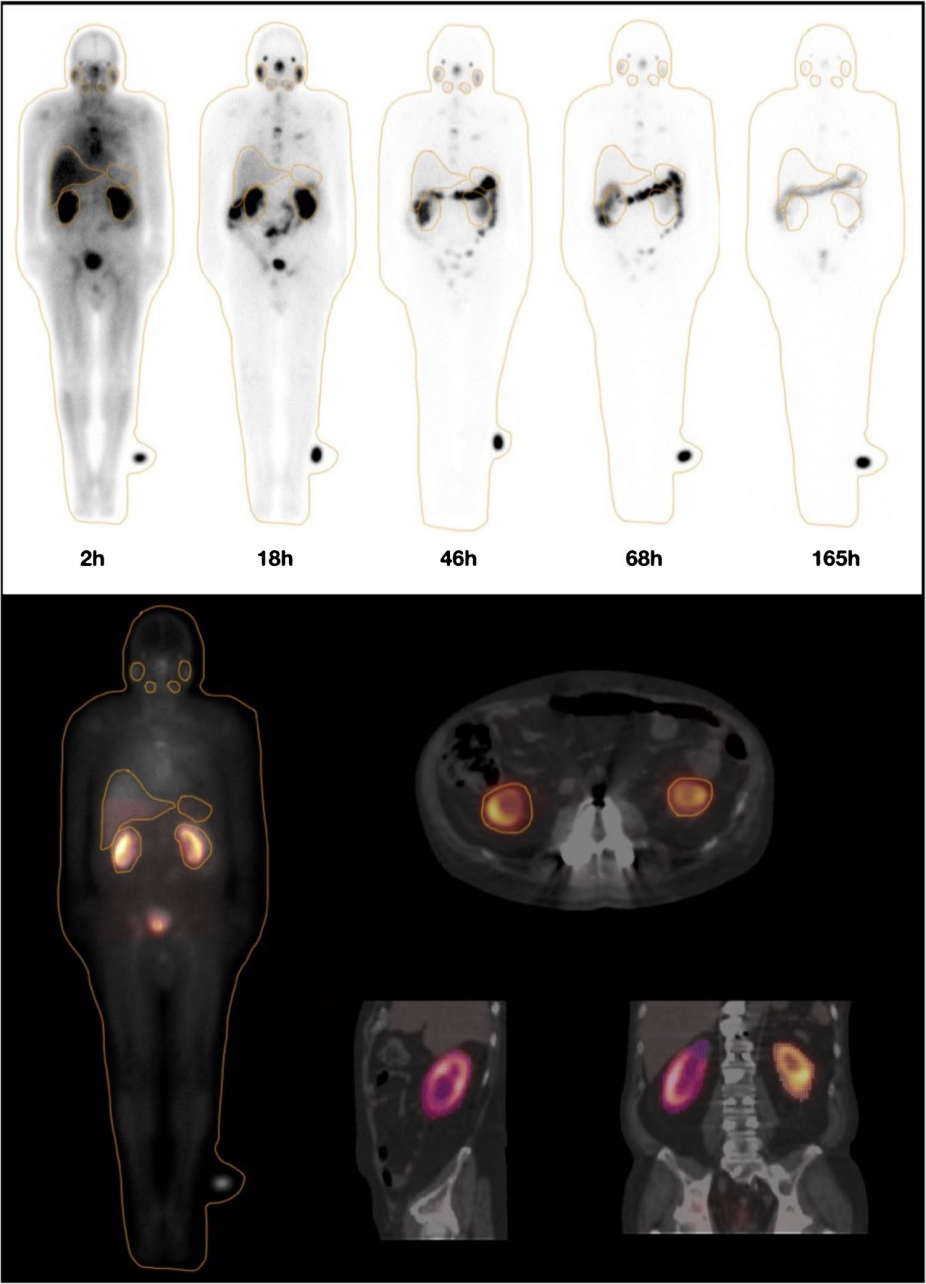
Planar whole-body images of five time points as well as one of the SPECT/CT images. Regions of interest were labeled on the liver, kidneys, spleen, parotid glands, submandibular glands, lacrimal glands, and bladder

We used Hermes [22] to calculate organ-level dosimetry. Hermes Medical Solutions (HERMES, Stockholm, Sweden) markets a suite of dosimetry tools, including Hybrid Viewer Dosimetry (HVD) and Olinda/EXM, which provides organ-level dosimetry calculation using medical internal radiation dose (MIRD) schema [23]. As shown in Fig. 2, planar whole-body images of five time points as well as one of the SPECT/CT images were loaded into HVD first, and targeted organs were then delineated by 2 board-certified nuclear medicine physicians. Time-integrated activity coefficient (TIAC) was performed by HVD, and residence time was input into the linked Olinda/EXM for dose calculation [24].

### Model setup

The dosimetry prediction model was developed with the input of both PET imaging features and clinical features, and the corresponding dosimetry of targeted organ as ground truth. During the training of the model, we tend to optimize the mean squared error loss

$$MSE=\frac{\stackrel{n}{\sum_{i=1}}({y}_{i}-\hat{{y}_{i}}{)}^{2}}{n}$$

where $\hat{{y}_{i}}$ is the predicted absorbed dose that the network assigns to the label $i$, ${y}_{i}$ is the ground truth of each input, and $n$ is the number of input data. ML techniques were recruited for the dose prediction. More information on the model setup is attached in the corresponding part of Supplementary material.

### Evaluation

To evaluate the prediction uncertainty of our ML-based model, we compared our estimations with population-based dose using mean absolute percentage error (MAPE). Population-based dose may be estimated empirically before the treatment based on published data. In this study, we considered the mean dosimetry results from population-based dosimetry estimations published previously [25] as a reference. We used a mean absorbed dose of 0.72 Gy/GBq for the kidney and 0.12 Gy/GBq for the liver and the absorbed dose for salivary glands (0.59 Gy/GBq) was averaged over the parotid and submandibular glands. Due to the absence of spleen dose [25], we adopted the average absorbed dose from our own dataset, which was 0.31 Gy/GBq.

## Results

### Time activity curve (TAC) and dosimetry

**Fig. 3:**
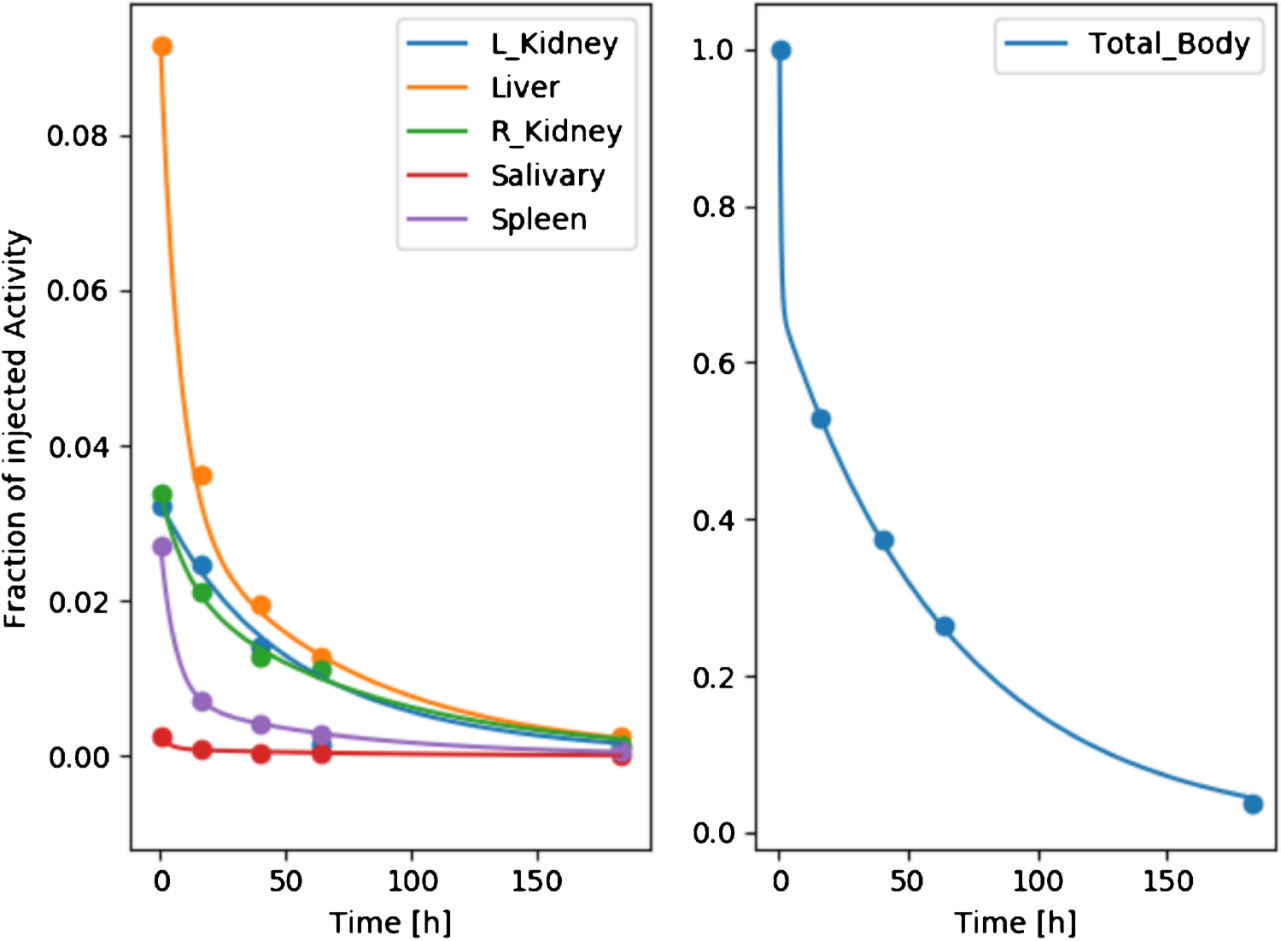
Example of time activity curve (TAC) generated by Hermes software

We used Hermes software to generate the TAC and absorbed dose of each target organ based on planar whole-body and SPECT/CT images. Figure 3 shows an example of a TAC, the left panel shows the change of fraction of injected activity over time of each organ, and the right panel shows the change of fraction of injected activity over time of the whole body. Complete statistics including absorbed dose for all subjects can be found in Table 2. According to both the TAC and Table 2, kidneys represent the critical organ with a mean absorbed dose of 0.65 Gy/GBq. Liver accounts for the largest percentage of cumulated activity in the entire body and decays at the fastest rate compared to any other organ. In contrast, salivary gland activity remained the smallest proportion of the whole body and decayed more slowly. All curves showed a similar trend of decay after 100 h. Additionally, Supplementary Fig. 4 shows that the greater time interval between each treatment cycle, the absorbed dose of each organ tended to be less correlated.

**Table 2:** Absorbed dose for all subjects of each organ as well as the whole body (in Gy/GBq)

Cycles investigated	Whole body	Kidneys	Liver	Salivary glands	Spleen
Overall (*n* = 43)					
Mean ± SD	0.031 ± 0.017	0.648 ± 0.165	0.067 ± 0.035	0.565 ± 0.389	0.306 ± 0.227
Range	0.012–0.078	0.236–1.041	0.019–0.151	0.150–1.869	0.033–0.918
First cycle (*n* = 21)					
Mean ± SD	0.031 ± 0.016	0.572 ± 0.167	0.060 ± 0.035	0.480 ± 0.269	0.231 ± 0.20
Range	0.012–0.078	0.236–0.820	0.019–0.145	0.150–1.047	0.039–0.715
Second cycle (*n* = 11)					
Mean ± SD	0.033 ± 0.023	0.676 ± 0.266	0.068 ± 0.030	0.596 ± 0.464	0.337 ± 0.395
Range	0.015–0.076	0.314–1.159	0.036–0.128	0.257–1.359	0.033–1.341
Third cycle (*n* = 5)					
Mean ± SD	0.038 ± 0.029	0.753 ± 0.219	0.079 ± 0.033	0.775 ± 0.739	0.570 ± 0.258
Range	0.017–0.059	0.514–1.041	0.048–0.123	0.257–1.869	0.320–0.918
Fourth cycle and further (*n* = 6)					
Mean ± SD	0.033 ± 0.008	0.614 ± 0.172	0.080 ± 0.044	0.602 ± 0.304	0.322 ± 0.272
Range	0.024–0.039	0.328–0.780	0.024–0.151	0.323–1.134	0.046–0.732

### Model performance

**Fig. 4:**
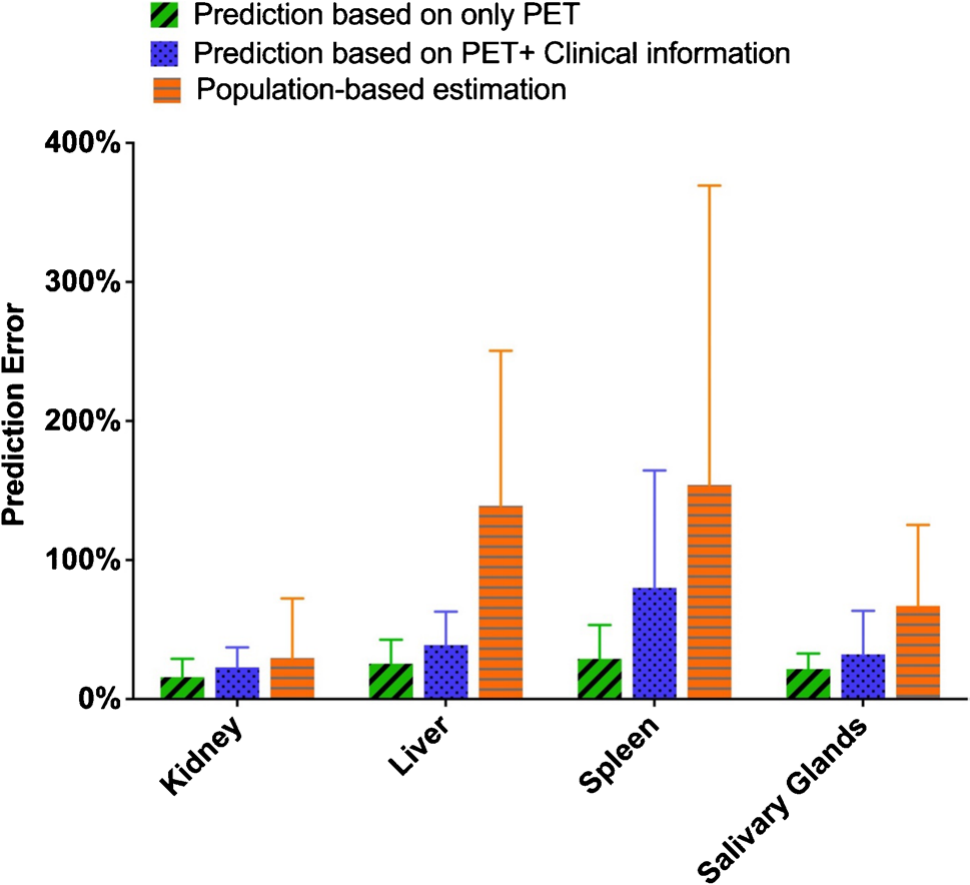
Comparison of prediction performance between individualized dose estimation with population-based model

Results of the comparison between ML-based dose estimation with population-based model are shown in Fig. 4. MAPE was used to assess the prediction uncertainty of estimated dosimetry. For kidney, the mean MAPE and standard deviation is 15.8% ± 13.2%. In contrast, population-based MAPE achieved 29.6% ± 42.8%. For the rest of the organs, the prediction of the liver, spleen, and salivary gland dosimetry achieved 25.5 ± 17.3%, 32.1 ± 31.4%, and 23.8% ± 13.1% with ML-based model, and the population-based MAPE is 139.1% ± 111.5% for the liver, 54.1 ± 215.3% for the spleen, and 67.0 ± 58.3% for the salivary glands. Paired *t* test showed significant difference between AI prediction and population-based estimation (*p* < 0.01). However, when combining PET imaging features with clinical blood test features, the prediction uncertainty of our model tends to increase in each target organ, probably for the reason that these features are non-organ-specific. More details of model performance can be found in the corresponding part of Supplementary material.

## Discussion

Dose plays a key role in the application of RLT. Current practice of PSMA-directed RLT applies fixed activity to patients. Similar to pharmaceutical practice, the efficacy and risk assessment generally follow the empirical experience [6]. The post-therapy dose distribution can be explicitly or implicitly estimated from cohorts of patients treated with the same protocol in previous studies. These empirical methods function well in clinical practice [26–28]. They enable efficient and economic RLT application and accelerate the clinical translation of novel RLT development. On the other side, RLT is still considered to be an approach to radiation oncology. The experience of external beam radiotherapy recommends the treatment planning to identify the optimal trade-off between therapeutic dose and side effects. Although treatment planning and dose application for RLT are generally recommended by regulatory agencies or scientific societies, no practical solution is available at the moment. Tumor sink effect in ^68^ Ga-PSMA-11 PET imaging was demonstrated previously [29]. These results suggest that candidates for PSMA-RLT with high tumor volume on pretherapeutic PSMA PET might benefit from increased therapeutic activity without exceeding the radiation dose limit for organs at risk.

Pretherapeutic dosimetry estimation is essential to the realization of treatment planning. However, the relationship between pre-therapy imaging and post-therapy dosimetry is complex. The pre-therapy SUV is a single time point uptake measurement after 1 h, while the post-therapy dosimetry is an integration of radiation time course of a similar but not identical tracer over several days. Furthermore, therapeutic tracers (^177^Lu-PSMA I&T) are injected at several-fold higher activity than imaging tracers (^68^ Ga-PSMA-11), and their ligands are similar but not identical. The theranostic principle of similar pharmacokinetics between the imaging and therapy tracers (PSMA-11 [30] and PSMA I&T [25]) enables the qualitative assessment of the post-therapy dose before the treatment [31]. Previous studies revealed that SUV values of pre-therapy imaging correlate with the post-therapy dose distribution [32, 33]. These correlations confirmed taking into account the pre-treatment information may assist the estimation of the post-therapy dosimetry [34] and reduce the possibilities of under- or over-estimation of different biodistributions [35]. Our preliminary results showed that ML can decrease the uncertainty of this pretherapeutic dose assessment compared with empirical population-based estimation. The developed ML model took multivariate input and learned quantitative principles from the training data with the regularizations of their underlying interrelations such as multi-organ relative relations [36], which can complement the missing data in prediction. Furthermore, the key question of dosimetry prediction is sort of implicit estimation of the biological half-lives of the therapy tracer from imaging. Previous studies have achieved single time point estimation by assuming that the tissue-specific radioligand uptake curves for different patients are identical [37, 38]. However, as shown in Supplementary Fig. 5, our results showed that the variation of these biological half-lives between individuals is although relatively small but not negligible (11.2 ± 6.0 h). Our data-driven ML approach modeled for each target organ will better consider the individual variation in the estimation of the absorbed dose. Additionally, Supplementary Fig. 6 shows that of the features extracted from pre-treatment PET imaging, SUV_max_ and tumor volume are most relevant to dose estimation.

Dosimetry methods have been established in the last decades to calculate the dose distribution of the applied therapeutic agents based on a series of planar or 3D images [39–47], which can be used to quantify the whole-body dosimetry of the therapeutic agent. The series of scintigraphy were taken at different time points to sufficiently cover the kinetics of the radiopharmaceutical. The Hermes tool recruited in our study was developed based on MIRD system [23, 39, 48], which is recommended by the European Association of Nuclear Medicine Dosimetry Committee Guidelines. In addition to a series of scintigraphy, we included at least one SPECT/CT for the reason of determining the overall calibration factor of the system sensitivity, which helped to convert the counts of scintigraphy (cts) to the activity concentration of Bq (per voxel). The recommendation of MIRD committee for calibration required extra phantom acquisitions [49], which was not feasible in our study. Inspired by Halty et al. [50], the fraction of activity in each organ was proposed to compute the calibration factor as the total number of counts in the SPECT image divided by this activity derived from the TAC.

There are several limitations of our study. The first is the inherent bias in the limited datasets, and the inclusion of additional subjects may further improve the generalizability and robustness of the developed model. Although the developed ML methods have demonstrated the potential to dissect the complex relation behind the correlation for dosimetry prediction, the limited data for training may limit its prediction power. Training and validation with additional data is necessary to improve the trustworthiness of the dose estimation based on ML. However, to the best of our knowledge, we are the first group to explore the feasibility of estimation of post-therapy dosimetry for ^177^Lu-PSMA I&T therapy; hence, the availability of these datasets is rather limited at this stage. For the second limitation, the *S* values applied by organ-level dose calculation tools like Olinda/EXM were obtained based on reference phantoms, which were not intended for individualized estimation, which would then result in the inclusion of inaccurate absorbed dose values in our ML model development. Potential uncertainty can occur both for volumetric assessment and SUV measurement for organs. Another limitation of our study is the absence of dose prediction for tumor lesions, which is due to the unavailability of the lesion phantom in the current version of Hermes tool. A similar problem occurred in the attempt to delineate the parotid and submandibular glands separately, which failed due to the absence of the corresponding phantom. We solved this problem by treating the parotid and submandibular glands as two parts of the salivary gland, which was provided as a phantom in Hermes.

## Conclusion

The preliminary results confirmed the feasibility of pretherapeutic dose estimation before the PSMA-RLT and its added value compared with empirical population-based estimation (*p* < 0.01); ML may decrease the estimation uncertainty of post-therapy dosimetry, with an average MAPE of 15.76% for critical dose-absorbing organs (i.e., kidneys). The exploration of dose prediction may support the identification of the role of treatment planning for RLT.

## Supplementary Information

Below is the link to the electronic supplementary material.

Supplementary file1 (DOCX 1552 KB)
