# Supplementary material for: Application of machine learning to pretherapeutically estimate dosimetry in men with advanced prostate cancer treated with 177Lu-PSMA I&T therapy
Source: Eur J Nucl Med Mol Imaging. 2022 Jun 30;49(12):4064–72. doi: 10.1007/s00259-022-05883-w (PMC9525373; doi:10.1007/s00259-022-05883-w)
Supplement: Supplementary file 1 — Supplementary file1 (DOCX 1552 KB) [file 259_2022_5883_MOESM1_ESM.docx]

**SUPPLEMENTARY MATERIAL**

**Application of Machine Learning to Pretherapeutically Estimate Dosimetry in Men with Advanced Prostate Cancer Treated with ^177^Lu-PSMA I&T Therapy**

**Model Setup**

**
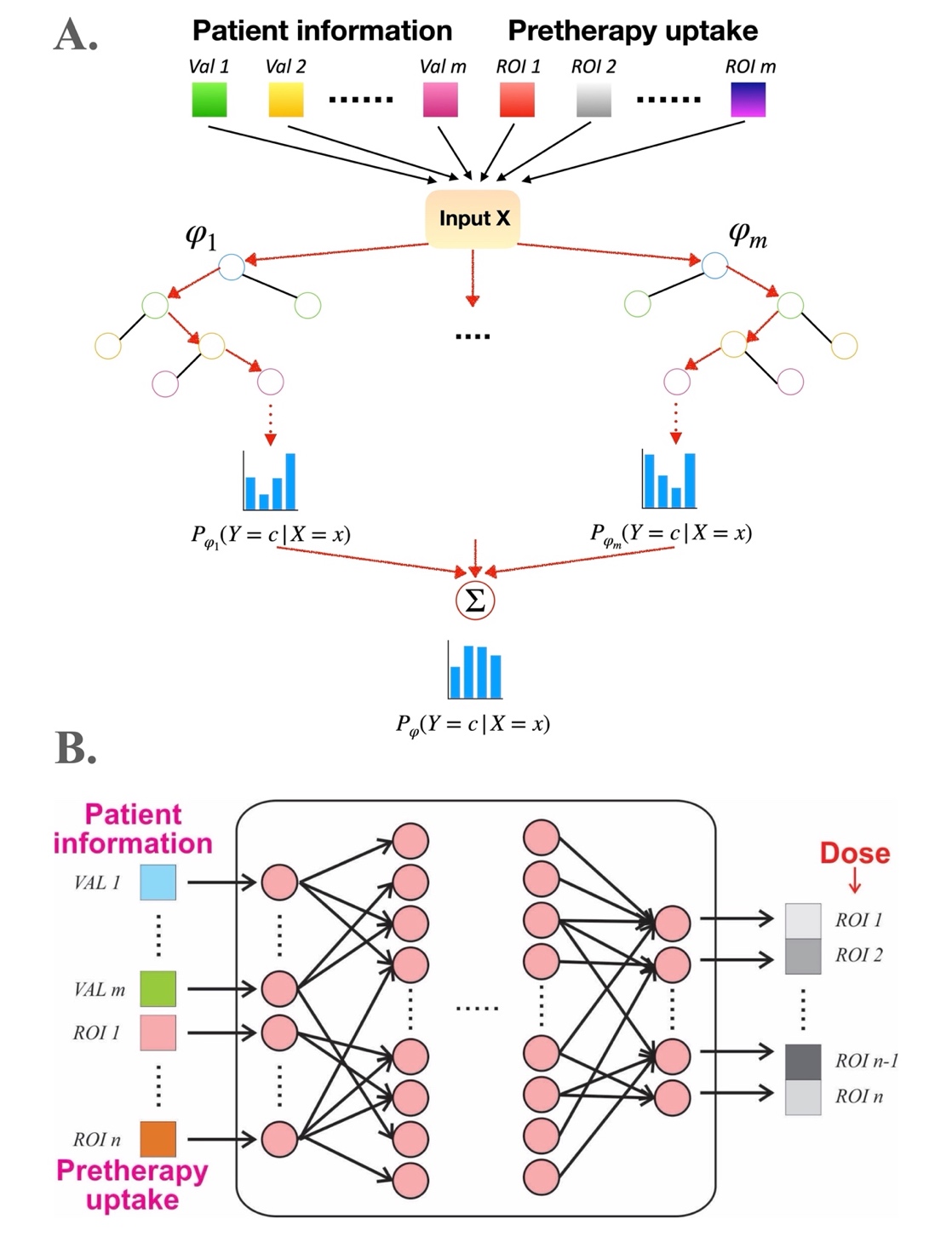
**

**Supplementary FIGURE 1.** Proposed Random Forest Regression (RFR) and artificial neural network (ANN).

Here, we proposed two approaches to solve this regression task, one is the traditional machine learning technique random forest regression (RFR) and the other is artificial neural network (ANN), which are both suitable for complicated regression tasks with high dimensionality. As shown in Supplementary Figure 1A, RF is bagged decision tree models that split on a subset of features on each split, trained recursively to minimize the error for each tree. ANN (Supplementary Figure 1B) consists of hundreds of artificial neurons and organized in layers, aiming to minimize the prediction error by optimizing interunit connections during training.

Random forest (RF) ensembles a bunch of regression tree and output the summed prediction of each tree, and trained recursively to minimize the Sum of Squared Error (SSE) for each tree,

$$SSE=\underset{c\in leaves(T)}{\sum}\underset{i\in c}{\sum}(y_{i}-m_{c})^{2}$$

*
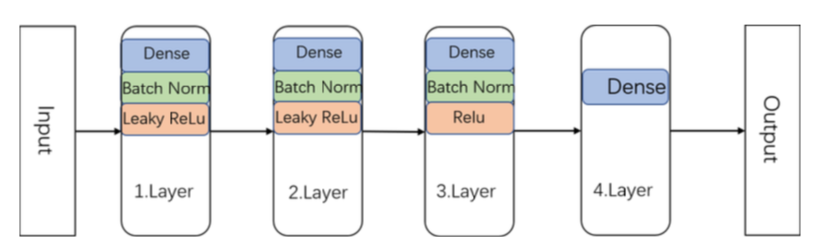
*where $m_{c}=\frac{1}{n_{c}}\underset{i\in c}{\sum}y_{i}$ is the prediction for leaf c. In our model the number of trees was set to 20, the minimum number of samples required to split an internal node is 2, the maximum depth of the tree is 5 layers, and the square root of total number of features were to consider when looking for the best split.

**Supplementary FIGURE 2.** Proposed artificial neural network (ANN) architecture.

As shown in Supplementary Figure 2, our proposed artificial neural network (ANN) is a 4-layer fully connected network. For the first three layers, batch normalization (BN) layer was added following the dense layer, which is used to normalize the input layer by adjusting and scaling the activations. Here all the layers use leaky rectified linear units (Leaky ReLU) as activation function except for the last, for the sake of the "dead" ReLU problem, in which case model always outputs the same value for any input. The last prediction layer was activated with Rectified Linear Units (ReLU). The network is trained end-to-end using gradient descent as optimization method. We train the model using batch size of 16, learning rate of 0.01 and pick the model with the lowest validation loss.

To further leverage the performance of our model, we applied feature selection before training. Techniques like forward selection, principal component analysis (PCA) and analysis of variance (ANOVA) of F-value were recruited in search of minimally sized but most relevant subset of input features, which not only boost model efficiency and interpretability, while reducing the risk of overfitting. Lasso linear regression is applied in forward selection model here and evaluated with mean accuracy.


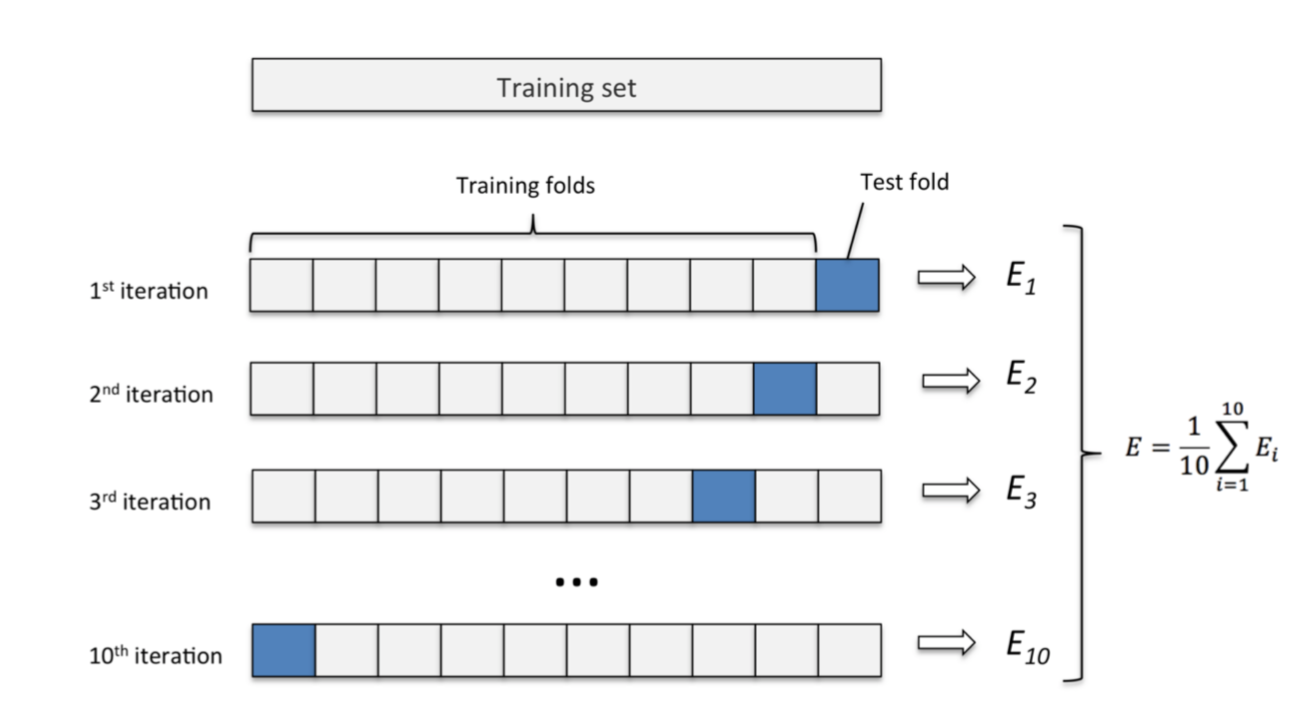
**Cross validation**

**Supplementary FIGURE 3.** K-fold cross validation.

We applied K-fold as our cross-validation method, which first divide data D into k folds of mutual exclusion subsets

$$D=D_{1}\cup D_{2}\cup\cdot\cdot\cdot\cup D_{k},D_{i}\cup D_{j}=\phi(i\neq j)$$

The consistency of data distribution will be remained for each subset $D_{i}$ by stratified sampling. One of the $k$ subsets was split as a hold out for test dataset each time of training, and the rest remained as a training dataset. Here we chose 10 as value of k.

**Time Activity Curve (TAC) and Dosimetry**


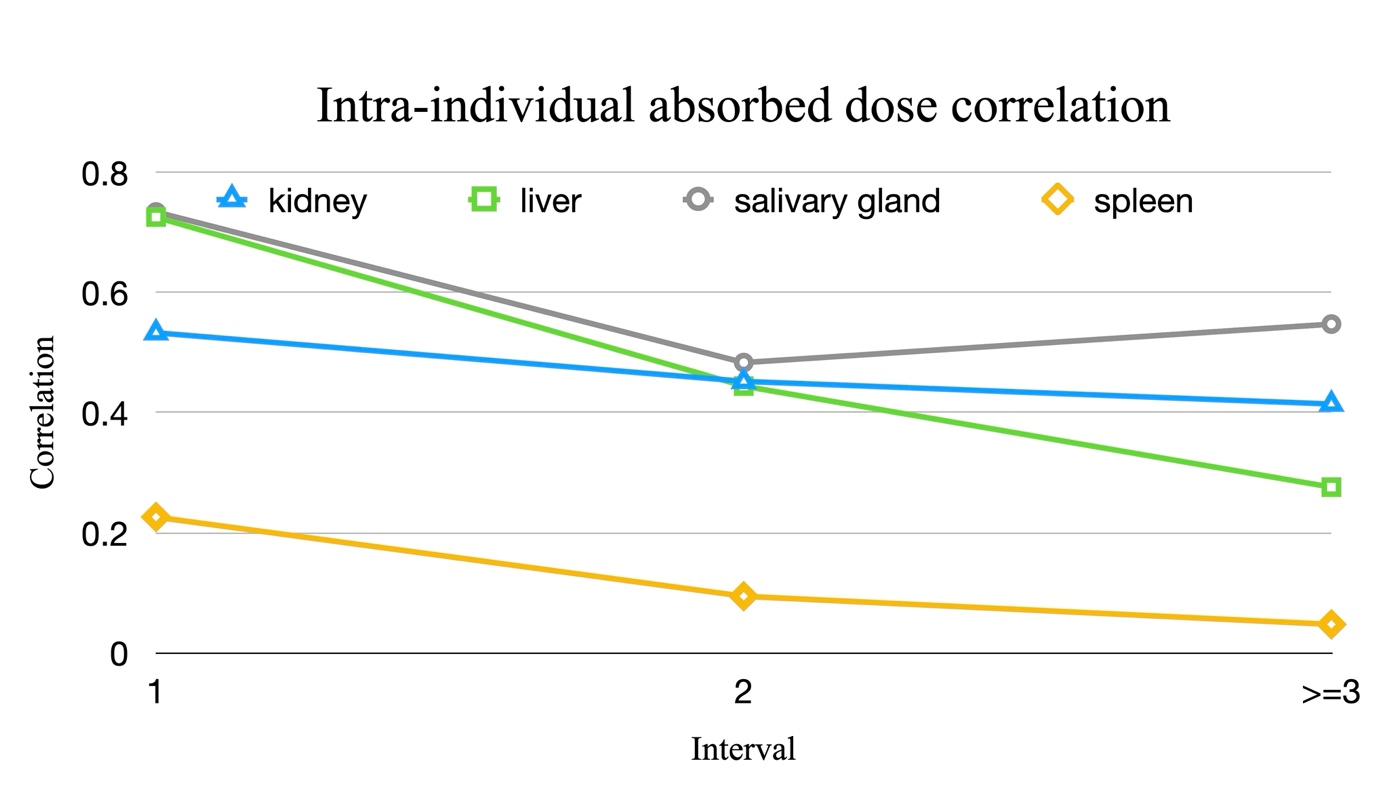


**Supplementary FIGURE 4.** Intra-individual correlation within patients who received multiple cycles of treatment.

**Supplementary FIGURE 5.** Variation of inter-individual biological total-body half-lives.

**Model Performance**


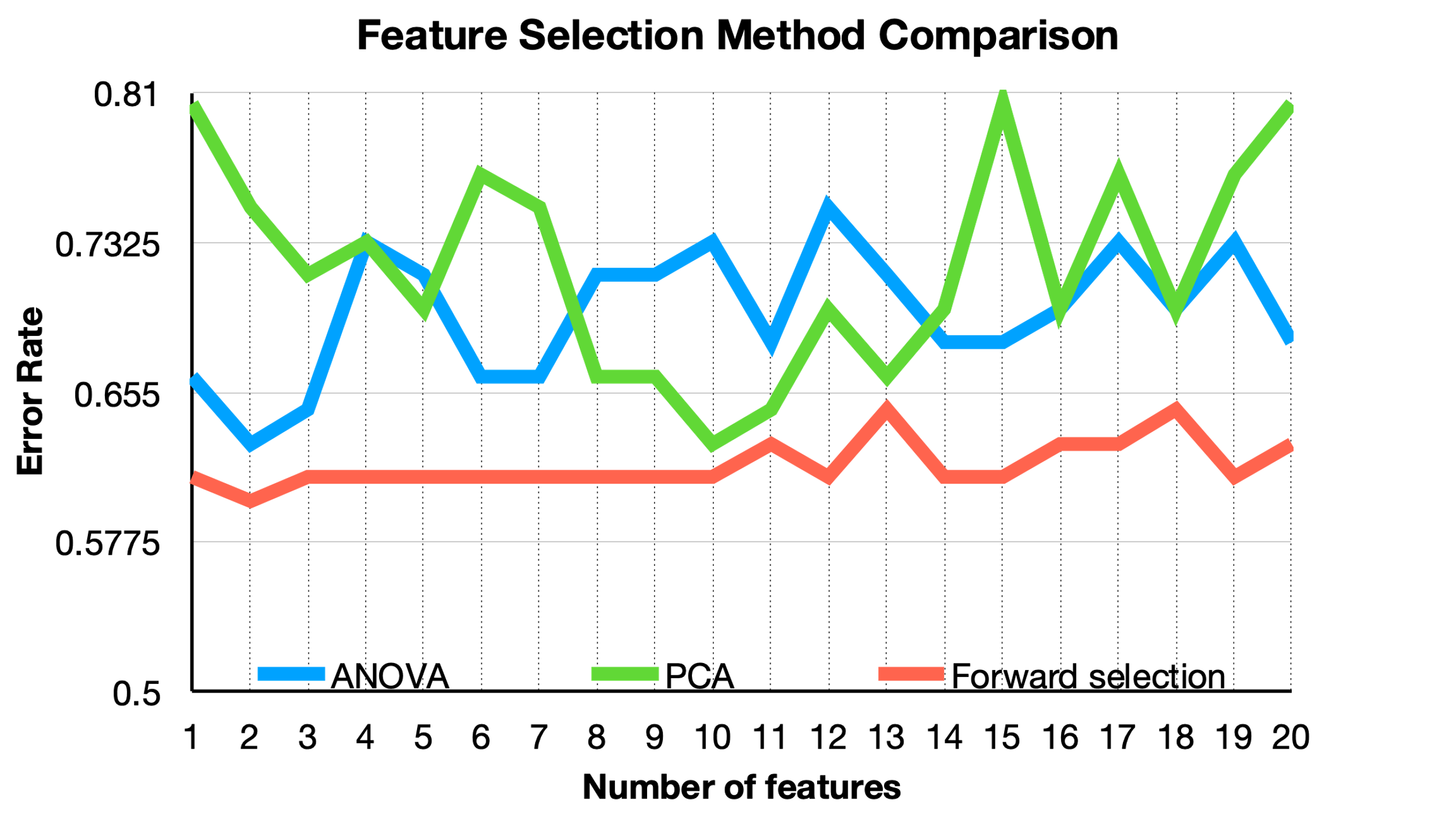


**Supplementary FIGURE 6.** The distribution of model accuracy along with number of features. The greedy forward selection method represented the best performance, topped at 0.599 of accuracy when 2 features are involved, which are SUV_max_ and Volume. Together with these two selected imaging features and clinical features, we trained and tested our ML model. We ensembled our ANN model and RFR model to achieve the best results in this comparison, it surpassed the population-based prediction in terms of every organ.


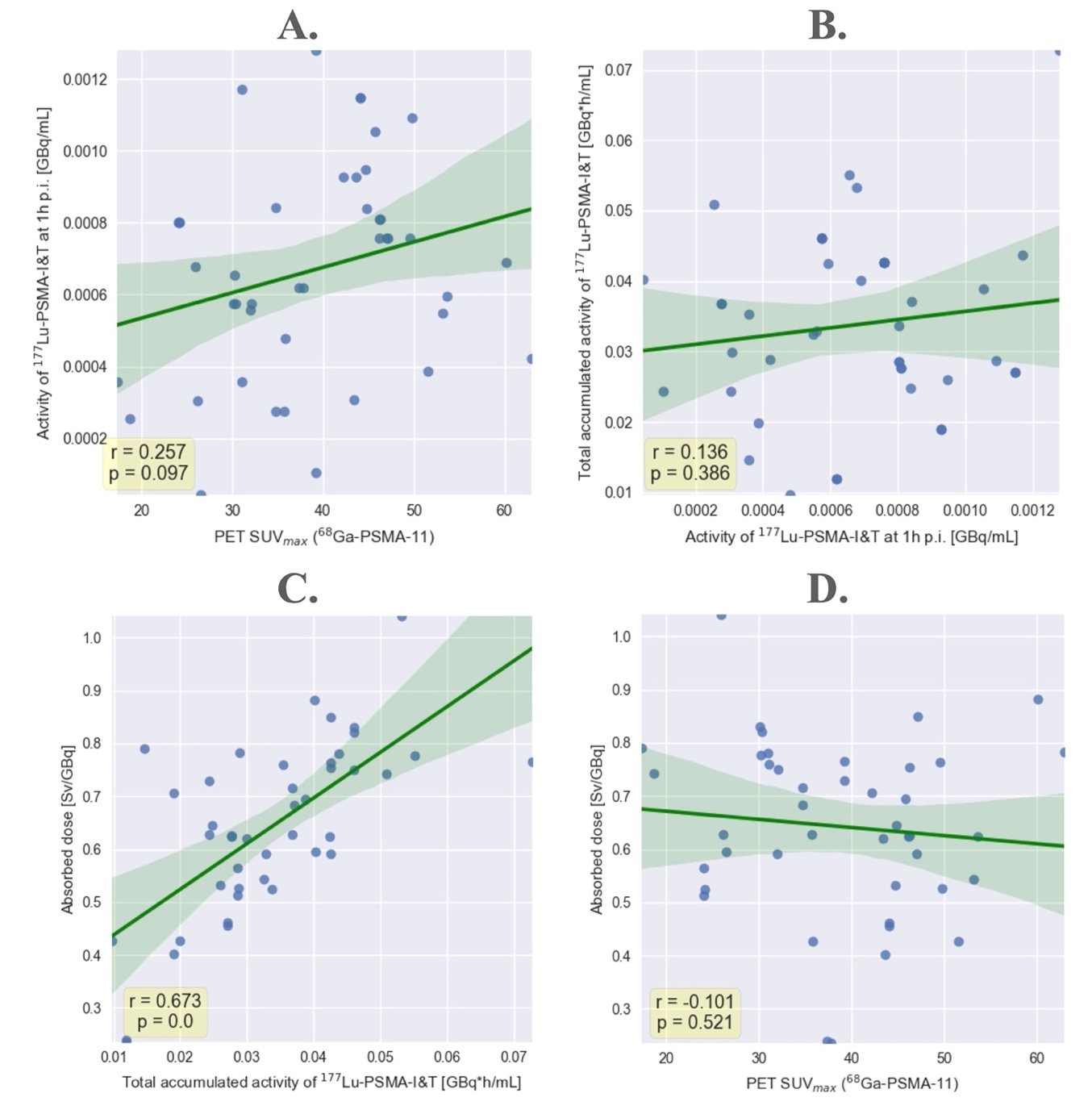


**Supplementary FIGURE 7.** The distribution of model accuracy along with increase of the number of features.

Moreover, we examined the relationship of each intermediate features, aiming to investigate the intrinsic mechanism of our model in this prediction task. Based on the parameters of TAC, we were able to simulate an exponential curve to obtain organ activity at specific post-inject time point, as well as total accumulated activity within one therapy cycle. Relevance plot of each intermediate features are shown in Supplementary Figure 5, here we presented the results of kidney as an example. The result indicates that total accumulated activity and absorbed dose are strongly relevant (p<0.1), while the relevance of other pairs tends to be weak.


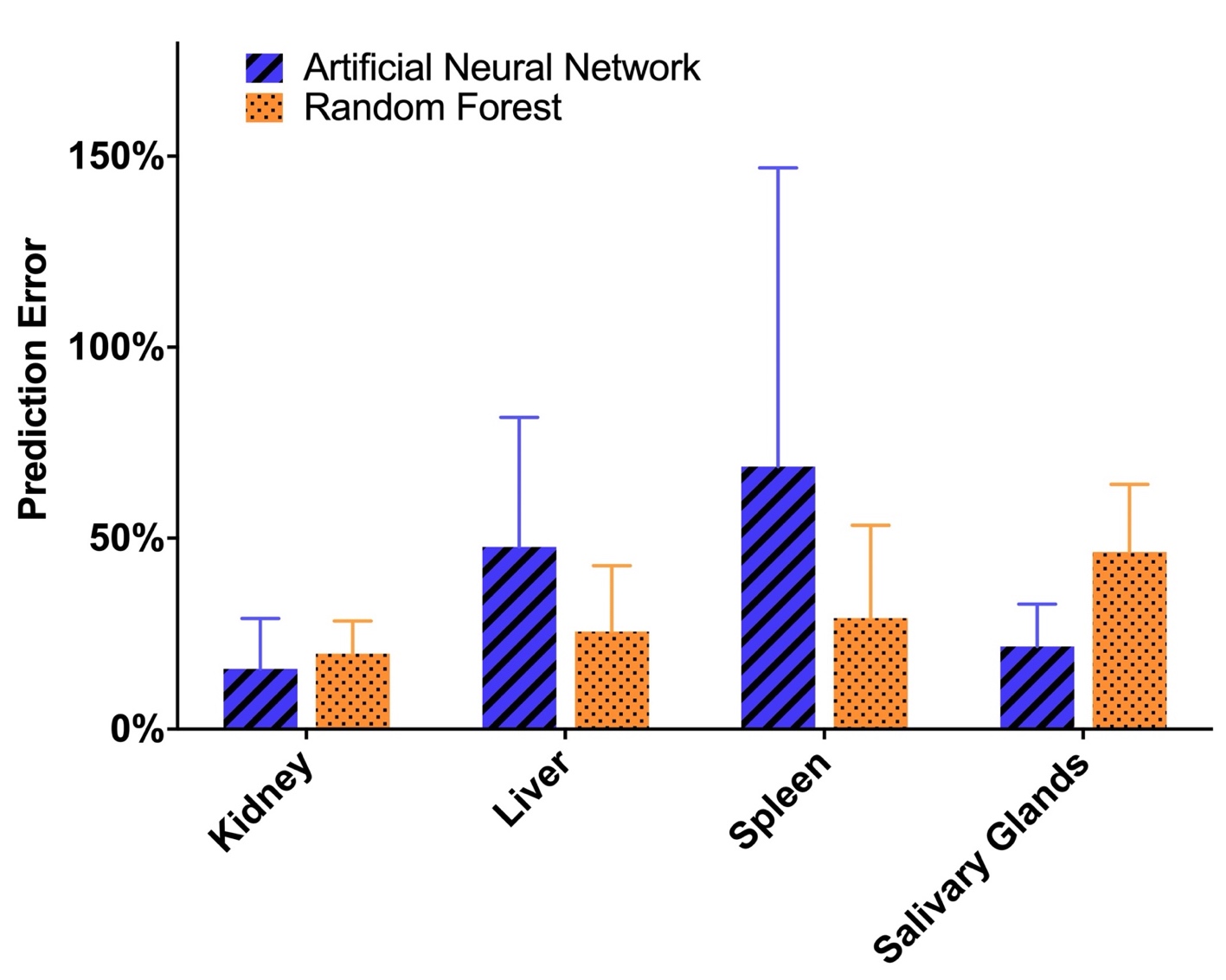


**Supplementary FIGURE 8.** The inner comparison of prediction performance between Random Forest Regression (RFR) and artificial neural network (ANN).

The inner comparison between ANN model and RFR model is shown in Supplementary Figure 6, it suggests that the RFR model appears to be a candidate for higher accuracy in dose prediction tasks, except for the kidney and salivary gland, for which a more complicated model is required simply because of the greater number of input features involved. This implies that RF model exhibit a so-called saturation curve of their attainable accuracies, which possibly indicates that as the amount of dataset grow rapidly in the future work, the performance of ANN might surpass the RF model, with its more complex and easily expandable structure.
